# EXERT‐BC: A pilot study of an exercise regimen designed to improve functional mobility, body composition, and strength after the treatment for breast cancer

**DOI:** 10.1002/cam4.7001

**Published:** 2024-03-16

**Authors:** David J. Carpenter, Chris Peluso, Christie Hilton, Frank Velasquez, Adam Annichine, Krista Matsko, Jared Rosenberg, Alexander K. Diaz, Parker Hyde, Sushil Beriwal, Colin E. Champ

**Affiliations:** ^1^ Department of Radiation Oncology Wellstar Paulding Medical Center Hiram Georgia USA; ^2^ Department of Radiation Oncology Duke University Medical Center Durham North Carolina USA; ^3^ Exercise Oncology Consortium Pittsburgh Pennsylvania USA; ^4^ Allegheny Health Network Cancer Institute Exercise Oncology and Resiliency Center Pittsburgh Pennsylvania USA; ^5^ Department of Medical Oncology Allegheny Health Network Pittsburgh Pennsylvania USA; ^6^ Allegheny Health Network Sports Performance Center Pittsburgh Pennsylvania USA; ^7^ Department of Exercise Science Syracuse University Syracuse New York USA; ^8^ Department of Radiation Oncology Murray‐Calloway County Hospital Murray Kentucky USA; ^9^ Department of Kinesiology Northern Georgia University Dahlonega Georgia USA; ^10^ Department of Radiation Oncology Allegheny Health Network Pittsburgh Pennsylvania USA

**Keywords:** body composition, breast cancer, exercise science, hypertrophy, resistance training

## Abstract

**Purpose:**

Resistance training may offer several unique advantages within breast cancer (BC) survivorship care; however, safety concerns have limited the application of high‐intensity compound movements necessary to elicit optimal changes in body composition, strength, and quality of life in this population. The EXERT‐BC trial assesses the safety and feasibility of an evidence‐based, dose‐escalated resistance training regimen among BC survivors, with the goal of improving physical and metabolic function, mobility, muscle mass, and body composition.

**Methods:**

Participants included women with breast cancer underwent a 3‐month thrice weekly exercise regimen involving dose escalation of high‐intensity compound exercises. Coprimary outcomes included safety and adherence. Pre‐ and post‐regimen assessment included body composition testing, functional mobility and balance, total load (weight × repetitions × sets) across compound exercises, and patient reported quality of life. Pairwise comparison was performed via the paired *t* test.

**Results:**

Fourty participants completed a 3‐month exercise regimen, with a median age of 57 years (range, 27–74 years) and 73% having stage 0–2 BC. BC therapies concurrent with exercise included anti‐estrogen therapy (80%), radiotherapy (30%), and non‐hormonal systemic therapy (15%). No adverse events were observed aside from a single case of self‐limited knee pain. Session attendance exceeded a prespecified threshold of 75%, and 98% patients reported ongoing compliance to an exercise regimen following regimen completion.

Significant reductions in percent body fat (*p* < 0.001) and increases in percent muscle mass (*p* = 0.011) were observed. Significant increases in resting metabolic rate (*p* = 0.023), bilateral grip strength (*p* < 0.001), functional movement screen (*p* < 0.001), bilateral Y‐Balance testing (*p* < 0.001), and Godin questionnaire scores (p < 0.001) were observed.

**Conclusion:**

A 3‐month dose‐escalated resistance training regimen comprising high‐intensity compound movements appears safe with a high degree of adherence among breast cancer survivors, resulting in demonstrable improvements in body composition, metabolic parameters, strength increases, and patient‐reported quality of life.

## Graphical abstract

Breast cancer survivors completing a 3‐month high‐intensity exercise regimen demonstrated significant improvements in exercise load across compound movements. Significant strength increases were accompanied by significant improvements across body composition, balance, functional mobility, and self‐reported quality of life.

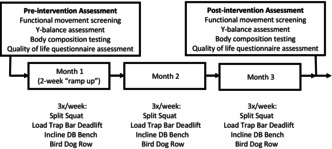

## 1 INTRODUCTION

The emerging field of exercise oncology reflects advances in both oncologic survivorship care and exercise science. Among breast cancer (BC) survivors, long‐term data across early stage and DCIS trial populations consistently demonstrate BC‐specific mortality rates under 5%,^1^, ^2^ highlighting the necessity of BC survivorship care extending beyond oncologic therapy. Risk factors for both BC incidence and unfavorable outcomes include obesity, decreased muscle mass, and decreased activity levels,^3^, ^4^, ^5^ Many BC survivors appear to fall short of daily activity level recommendations, prompting efforts to quantify and increase activity levels during and after treatment.^6^

Among oncologic survivors, sarcopenia is associated with decreased survival and increased treatment‐related toxicity.^7^ Accordingly, oncologic exercise programs should seek to optimize body composition parameters. Many exercise oncology interventions for BC survivors have prioritized cardiovascular and aerobic exercise over traditional resistance training.^8^ Advantages of an aerobic exercise program may include decreased requirements for resources and supervision. However, a meta‐analysis among non‐oncologic populations suggests that aerobic exercise results in limited fat loss in the absence of other lifestyle changes,^9^ and may be accompanied by corresponding losses in muscle tissue.^10^ These data appear consistent with randomized data among BC survivors that demonstrate no change in muscle mass from aerobic exercise.^11^ In contrast, resistance training may be of specific benefit to BC survivors due to improvements across fat loss, hypertrophy, bone mineral density, insulin sensitivity, and resting metabolic rate among other metabolic parameters.^12^, ^13^ Such well‐documented improvements across non‐oncologic populations warrant further evaluation among BC survivors.

Rigorous evaluation of resistance training among BC survivors requires careful adherence to evidence‐based principles. As with any other oncologic intervention, the efficacy of resistance training varies greatly depending on the technique employed and treatment intensity. Studies in non‐oncologic populations show optimal increases across body composition and functional performance parameters when employing high‐intensity compound exercises involving multiple muscle groups that mirror functional movement patterns across regimens that emphasize linear progression of exercise weight, repetitions and/or sets (i.e., “dose escalation”).^14^, ^15^ Furthermore, non‐oncologic reports suggest that an exercise intensity of ≥80% of the maximum resistance tolerated for a single repetition (1RM) is optimal for overcoming the physiological thresholds that dictate muscle repair, bone remodeling, and resting metabolic rate among other parameters.^16^, ^17^, ^18^, ^19^, ^20^ In contrast, reports of resistance training in both general and BC populations, when limited to low intensity exercises (defined as exercises limited to repetitions 1RM <60%^17^) involving a small number of isolated muscle groups, have demonstrated minimal changes in body composition and functional performance.^21^, ^22^, ^23^

Despite the specific advantages that BC survivors may derive from resistance training, safety concerns have limited successful implementation of dose‐escalated resistance training of sufficient intensity. Exercise scientists and physical therapists who routinely prescribe resistance training in accordance with National Strength and Conditioning Association (NSCA) guidelines may be less familiar with medical clearance necessary for oncologic patients, while oncologists may have minimal context for counseling their patients regarding evidence‐based resistance training. To date, the authors are unaware of any studies implementing a dose‐escalated resistance training regimen involving high‐intensity compound movements in an oncologic population. Accordingly, the EXERT‐BC protocol represents a multidisciplinary effort involving practitioners with formal training in both oncology and resistance training, designed to assess the safety and feasibility of a resistance training regimen using high‐intensity compound exercises among BC survivors, with the goal of improving physical and metabolic function, mobility, muscle mass, and body composition.

## 2 METHODS

### 2.1 Participants

This institutional review board‐approved (Allegheny Health Network Review Board) single‐arm phase I trial (protocol 2022‐269‐SG) was registered at ClinicalTrials.gov (NCT NCT05747209) and conducted in accordance with CONSORT guidelines.^24^ Eligible patients included women aged 20–89 years with breast cancer or ductal carcinoma in situ (DCIS) who were deemed able to participate in an exercise regimen by a practitioner with dual medical doctor (MD) and Certified Strength and Conditioning Specialist (CSCS) certification. Mobility requirements included the ability to get up and down from the ground, as well as the ability to perform a single body weight squat (i.e., to bend at the knees ≥90 degrees while lowering the midsection). Concurrent receipt of radiation therapy and/or non‐cytotoxic systemic therapy for breast cancer was permitted, with no restrictions based on the nature, duration, time interval, or residual sequelae of any prior oncologic treatment. Exclusion criteria included severe arthritic, joint, musculoskeletal, or cardiovascular comorbidities that would preclude safe resistance training. Concurrent receipt of radiation therapy and/or systemic therapy for breast cancer was permitted, excluding cytotoxic chemotherapy. Trial enrollment was completed between September 15, 2022 and April 13, 2023 at a single academic tertiary care center, where patient screening was performed by study personnel at time of consultation or follow up with a surgical, medical, or radiation oncologist. Consent was obtained for each participant.

### 2.2 Exercise regimen

**FIGURE 1:**
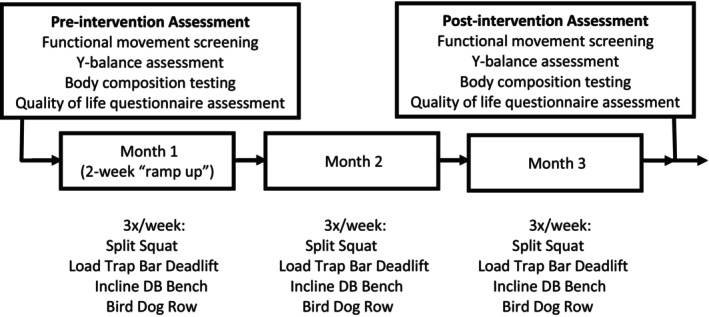
Exercise intervention. See Data S1 for a detailed example of an individualized exercise regimen.

Participants were enrolled to a 3‐month exercise regimen involving thrice weekly sessions of 45–60 min duration (Figure 1). All exercise sessions were performed at Health Network Exercise Oncology and Resiliency Center, which is a state‐of‐the‐art 3000 square foot exercise and research facility where exercise regimens are designed and supervised by CSCS utilizing exercise principles to improve strength, conditioning, performance, and overall health in accordance with NSCA guidelines.^16^ To minimize injury risk, each session was initiated by activation and reset exercises to optimize mobility, muscle activation, and range of motion. Afterward, to further maximize safety, compound exercises involving basic movement patterns of push, pull, hip hinge, squat, and core activation were completed prior to less intense, more isolated exercises. The initial 2 weeks of the regimen was considered a “run‐in” period, assessing strength and movement to guide selection of specific exercises and corresponding resistance. For example, if an individual was unable to split squat body weight, she would be assisted in the movement until able to progress to the weighted lift. Group sessions kept ratios of individuals to CSCS less than 6:1 to ensure safety.

Weight lifted (kg), repetitions, and sets performed were recorded, with load was calculated throughout (load = weight lifted × repetitions × sets). Regimens were designed to incorporate approximately 10 sets per muscle group per week as a threshold for promoting hypertrophy.^25^ Each daily workout progressed from a compound, multi‐joint movement to more isolated exercises. The multi‐joint movement was linearly progressed each month (i.e., increased resistance and/or sets as tolerated) to maximize strength and bone loading, while the other exercises were employed to improve strength, hypertrophy, and conditioning. To guide dose escalation of both exercise resistance (kg) and volume (number of repetitions and/or sets) across sessions, the supervising CSCS assessed repetition speed, number “left in the tank,” and rating of perceived exertion (RPE). In an effort to minimize the risk of overestimating longitudinal increases in exercise load due to early adaptation to exercise movement patterns during month one, it was determined a priori to analyze exercise load using measurements from the fourth week of each month. See the Data S1 for a detailed example of the program.

Attendance was recorded for all patients at each assigned session. Remote sessions were permitted only after complete assessment during the first month of the regimen, for which patient attendance necessitated documentation of weights utilized, sets, and repetitions using similar exercise equipment to that of the group session. Co‐primary endpoints included regimen safety and adherence, with an a priori threshold of 75% attendance across all sessions. Compliance was defined as an individual attending 75% of all sessions. Secondary endpoints included changes in body composition (i.e., percent body fat and muscle mass), functional mobility and balance, resting metabolic rate (RMR), patient‐reported quality of life, and post‐intervention activity levels.

### 2.3 Assessments

Prior to both exercise regimen initiation and upon regimen completion, body composition parameters were obtained via two methodologies at the adjacent Exercise Oncology Lab. First, an InBody 970 bioimpedance analysis (BIA) machine (InBody Co., South Korea) was used to quantify body fat (kg, percentage), muscle mass (kg percentage), fat‐free mass (kg, percentage), RMR (kcal), and phase angle. InBody testing offers a noninvasive means of quantifying body composition via bioelectric impedance analysis to precisely measure body composition without exposure to ionizing radiation. Phase angle reflects cellular membrane function, and has been shown to correlate with oncologic outcomes.^26^ Second, ultrasound (US) testing was performed concurrently as an additional means of quantifying body fat (kg, percentage), fat‐free mass (percentage), and RMR (kcal) via Body Metrix software (BodyMetrix, Brentwood, CA) performing the Jackson & Pollack calculation from US measurements at the triceps, suprailiac, abdominal, and thigh area.^27^

To assess functional movement and balance prior to and following the exercise regimen, participants underwent a seven movement Functional Movement Screen (FMS; scored as 0–21) and Y‐balance test ([the sum of 3 reach directions]/3 × limb length (cm) × 100). A Jamar Hand Dynamometer grip strength measurement device was used to measure grip strength before and after regimen completion. Participants completed EQ‐5D‐5L and Godin Leisure‐Time Exercise Questionnaires before and after regimen completion. The EQ‐5D‐5L is a five question quality of life survey with answers ranging in severity from no problem to inability to complete a task, with questions assessing mobility, self‐care, usual activities, pain/discomfort, and anxiety/depression. The sixth question is a self‐score of overall health from 0 to 100. This survey has been found to be short and easy with high accuracy, strongly correlating with quality of life.^28^ Godin questionnaires quantifies scoring to provide a Leisure Score Index light, moderate, and strenuous exercise to result in a score of >24 for active, 14–23 for moderately active, and <14 for insufficiently active.

Safety was continually assessed during each workout regimen by the exercise physiologist, with corresponding adjustments to exercise performance during each individual workout. Common Terminology Criteria for Adverse Events (CTCAE) version 5.0 was utilized to assess adverse events, and these were recorded in the subject's record and reported to safety oversight for summary reporting of the study's objective.

Anthropometric, metabolic, fitness, and quality of life measurements were analyzed as continuous variables. Pairwise comparisons were assessed via the paired *t* test. Missing data, compromising a single set of baseline grip strength measurements and single set of 3‐month exercise load calculations, were excluded from corresponding pair‐wise analyses. All statistical analyses were performed using R version 4.1.2 (R Project for Statistical Computing).

## 3 RESULTS

**FIGURE 2:**
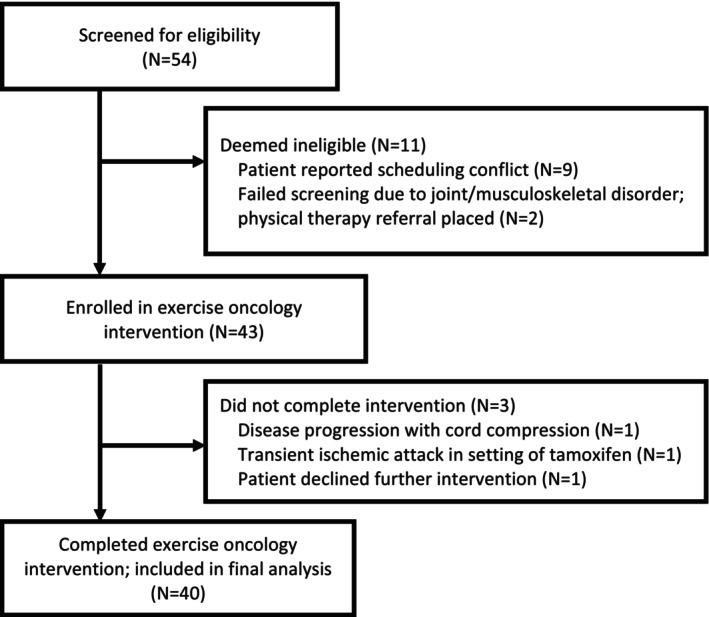
Consort diagram.

A total of 54 patients were screened for eligibility (Figure 2). Nine patients chose not to participate due to scheduling conflicts. During pre‐regimen screening, two patients (4%) were referred to physical therapy due to joint and musculoskeletal conditions precluding safe resistance training. Forty of 43 (93%) enrolled patients completed the exercise regimen and were thus included in analysis. Reasons for regimen withdrawal included the following: breast cancer progression with cord compression diagnosed clinically at the exercise facility (*n* = 1), transient ischemic attack presumed secondary to ongoing tamoxifen use (*n* = 1), and voluntary withdrawal in the absence of a documented adverse event (*n* = 1; 2%). Enrollment took place from September 16, 2022, to April 13, 2023.

Median patient age at time of enrollment was 56.5 years, with 17 patients (43%) over 60 years and 4 (10%) over 70 years of age (range, 27–74 years; Table 1). Nine patients (23%) endorsed a prior history of exercise within the past 6 months defined as exercise greater than walking and with or without resistance training. A majority of BC patients had DCIS or early stage disease (73%). Median time from initial BC diagnosis to study enrollment was 15 months, with 21/44 individuals starting 3 months after diagnosis. Concurrent oncologic therapies during the exercise intervention included anti‐estrogen therapies (80%), radiotherapy (30%), and non‐hormonal systemic therapies (12.5%; abemaciclib, *n* = 1; trastuzumab, *n* = 1; trastuzumab deruxtecan, *n* = 1; trastuzumab emtansine, *n* = 1; pembrolizumab, *n* = 1).

**TABLE 1:** Patient demographics and breast cancer characteristics.

	*N*	%
Median Age at Enrollment (Interquartile range)	56.5	(51–64)
Prior History of Exercise	9	23
Breast Cancer Stage		
*Ductal carcinoma in situ*	3	8
*Early stage*	26	65
*Locally advanced*	8	20
*Locoregional recurrence*	2	5
*Distant metastases present*	1	3
Lymphedema at Time of Enrollment	7	18
Concurrent Receipt of Anti‐Estrogen Therapy	32	80
Concurrent Receipt of Non‐Hormonal Systemic Therapy^a^	5	12.5
Concurrent Radiotherapy	12	30

^a^ Concurrent therapies: Abemaciclib, Trastuzumab deruxtecan, Trastuzumab emtansine, Trastuzumab, pembrolizumab.

Among those completing the exercise regimen (*n* = 40), a mean of 2.20 session absences (range, 0–10 sessions) were documented, including a mean 0.79 unexcused absences (range, 0–7 sessions). Overall adherence was under 75% in one individual, with attendance at 72.2%. Remote session attendance was utilized for 3 patients (8%), each for a single week while traveling out of town. No exercise‐related adverse events were documented aside from a single patient (2%) endorsing a 2‐day duration of knee soreness, which fully resolved with conservative measures. Of 7 patients (18%) endorsing lymphedema at time of enrollment, 1 (14%) reported improved lymphedema. Irrespective of baseline lymphedema status, no patients endorsed new or worsening lymphedema.

Pre‐ and post‐intervention values for body composition and quality of life parameters are provided in Table 2. While no significant differences in weight (kg) or absolute body fat (kg) were observed by InBody or US measurement, pairwise comparison involving both methodologies demonstrated significant reductions in percent body fat (InBody, 38.9% [interquartile range (IQR), 33.4%–44.1%] to 36.1% [IQR, 30.5%–43.5%], *p* < 0.001; US, 37.4% [IQR, 33.0%–39.6%] to 32.6% [IQR, 28.2%–37.8%], *p* < 0.001). Concurrent, significant increases were observed for InBody percent muscle mass (33.8% [IQR, 30.2%–36.0%] to 35.8% [IQR, 31.9%–40.0%], *p* = 0.011) and US percent fat‐free mass (29.0% [IQR, 26.0%–30.7%] to 29.4% [IQR, 27.6%–32.2%], *p* < 0.001). Across both InBody and US measurements, significant increases were observed for resting metabolic rate (InBody: 1366 kcal [IQR, 1306–1454 kcal] to 1382 kcal [IQR, 1306–1467 kcal], *p* = 0.023; US: 1403 kcal [IQR, 1324–1483 kcal] to 1437 kcal [IQR, 1352–1540 kcal], *p* < 0.001). A significant increase in body phase angle was also observed (4.9 degrees [IQR, 4.6–5.2 degrees] to 5.1 degrees [IQR, 4.8–5.4 degrees], *p* < 0.001). Significant increases were observed across bilateral grip strength, both overhead (right 18 kg [IQR 15–22] to 24 kg [IQR 20–28], *p* < 0.001, and left 20 kg [IQR 16.8–22.3] to 26 kg [IQR 22–28.5], *p* < 0.001) and at waist height (right 21.5 kg [IQR 18.8–26.3] to 26 kg [IQR 23–28.5], *p* < 0.001, left 20 kg [IQR 17.8–24.3] to 27 kg [IQR 22–29.5], *p* < 0.001).Significant increases were also observed across functional movement testing (9, IQR [8–11] to 12, IQR [10–14], *p* < 0.001), and bilateral Y‐Balance testing (Left: 72.3 [IQR, 63.9–81.2] to 83.9 [IQR, 78.3–98.1], *p* < 0.001; Right: 73.0 [IQR, 62.3–80.2] to 86.2 [IQR, 76.5–95.0], *p* < 0.001).

**TABLE 2:** Body composition and quality of life parameters before and after exercise intervention.

	Baseline (median [IQR])	Post‐intervention (median [IQR])	*p* Value
Height (inch)	64.8 (63–66)	–	N/A
Weight (initial)	168.7 (148.3–192.9)	163.1 (142.9–191.2)	0.067
InBody Body Fat (kg)	30.7 (21.4–40.0)	28.2 (19.4–40.9)	0.548
InBody Body Fat (%)	38.9 (33.4–44.1)	36.1 (30.5–43.5)	<0.001
US Body Fat (%)	37.4 (33.0–39.6)	32.6 (28.2–37.8)	<0.001
US Essential Fat (kg)	24.5 (20.3–27.4)	24.9 (18.1–28.3)	0.978
US Excess Fat (%)	6.6 (0.0–18.9)	2.8 (0.0–13.4)	0.002
InBody Muscle mass (kg)	25.0 (23.4–27.9)	25.6 (23.6–28.0)	0.103
InBody Muscle Mass (%)	33.8 (30.2–36.0)	35.8 (31.9–40.0)	0.011
InBody fat Free Mass (kg)	45.9 (43.0–50.4)	46.9 (43.3–50.8)	0.0867
US Fat‐free Mass (%)	29.0 (26.0–30.7)	29.4 (27.6–32.2)	<0.001
InBody RMR (kcal)	1365.5 (1305.5–1453.8)	1381.5 (1306.3–1467.3)	0.023
US RMR (kcal)	1403 (1324–1482.8)	1437 (1352.3–1540)	<0.001
Bone Mineral Content (kg)	2.8 (2.6–3.0)	2.8 (2.6–3.0)	0.349
Whole Body Phase Angle (degrees)	4.9 (4.6–5.2)	5.1 (4.8–5.4)	<0.001
Right Grip Strength, Overhead (kg)	18 (15–22)	24 (20–28)	<0.001
Right Grip Strength, at Waist (kg)	21.5 (18.8–26.3)	26 (23–28.5)	<0.001
Left Grip Strength, Overhead (kg)	20 (16.8–22.3)	26 (22–28.5)	<0.001
Left Grip Strength, at Waist (kg)	20 (17.8–24.3)	27 (22–29.5)	<0.001
Functional Mobility Screen Score	9 (8–11)	12 (9.8–14)	<0.001
Y‐Balance Test Score, Left Side	72.3 (63.9–81.2)	83.9 (78.3–98.1)	<0.001
Y‐Balance Test Score, Right Side	73.0 (62.3–80.2)	86.2 (76.5–95.0)	<0.001
EQ5D1 Score	5 (5–5)	5 (5–5)	N/A
EQ5D2 Score	5 (5–5)	5 (5–5)	N/A
EQ5D3 Score	5 (5–5)	5 (5–5)	N/A
EQ5D4 Score	4 (4–5)	4 (4–5)	N/A
EQ5D5 Score	5 (4–5)	5 (4.8–5)	N/A
EQ5D6 Score	73.5 (60–86.3)	85 (70–95)	<0.001
Godin Questionnaire Score	23.5 (6–35.3)	41.5 (32.8–55.5)	<0.001

Abbreviations: IQR, interquartile range, EuroQol 5 Dimension Questionnaire;kcal, kilocalories; kg, kilograms; RMR, resting metabolic rate; US, ultrasound.

Given the skewdness of scores for EQ5D1–5 responses (all parameters with lower 25% interquartile of responses ranging from 4 to 5 on a 5‐point scale), pairwise comparisons were not performed. However, EQ5D6 scores (0 to 100 recorded by an individual for their current overall health‐related quality of life) were significantly greater following an exercise regimen (74 [IQR, 60–86] to 85 [IQR, 70–95], *p* < 0.001), as were Godin questionnaire scores (24 [IQR, 6–35] to 42 [IQR, 33–56], *p* < 0.001).

Total exercise load (kg × repetitions × sets) was calculated across compound exercises during the final week of months 1, 2, and 3 (Table 3), with significant increases observed in pairwise comparison of month 1 to 3 values across split squat (192 kg [IQR, 15–299 kg] to 383 kg [IQR, 327–585 kg], *p* < 0.001), trap bar deadlift (1131 kg [IQR 912–1298 kg] to 1415 kg [IQR, 1012–1667 kg], *p* < 0.001), incline dumbbell bench press (170 kg [IQR, 136–204 kg] to 272 kg [IQR, 265–352 kg], *p* < 0.001), and bird dog row exercises (204 kg [IQR, 136–228 kg] to 340 kg [IQR 299–374 kg], *p* < 0.001).

**TABLE 3:** Total load across compound exercises at the end of months 1, 2, and 3.

	Load (kg × Repetitions × Sets)			
Exercise	Month 1, Week 4	Month 2, Week 4	Month 3, Week 4	*p* Value, Month 1 vs 3
Load Split Squat	192 (15–299)	383 (268–479)	383 (327–585)	<0.001
Load Trap Bar Deadlift	1131 (912–1298)	1210 (944–1527)	1415 (1012–1667)	<0.001
Incline Dumbbell Bench	170 (136–204)	261 (204–278)	272 (265–352)	<0.001
Bird Dog Row	204 (136–228)	272 (204–310)	340 (299–374)	<0.001

*Abbreviation*: kg = kilograms.

At 12 weeks following completion of the 3‐month exercise intervention, 39 of 40 (98%) patients reported ongoing compliance to an exercise regimen.

## 4 DISCUSSION

The present trial reports a novel dose‐escalated resistance training regimen employing high‐intensity compound exercises among BC survivors, demonstrating a promising safety profile and high rates of exercise adherence during and after the prescribed regimen. Moreover, significant improvements were observed in body composition including decreased body fat and increased muscle mass, increased resting metabolic rate, increased muscular strength, improved balance and functional mobility, and improved patient‐reported quality of life. These data represent a multidisciplinary effort spanning exercise science and oncologic survivorship, and may provide a template for further exercise oncology efforts that aim to optimize quality of life among BC survivors.

Significant increases in loads lifted were uniformly seen in the participants, which is in line with other studies in non‐cancer populations utilizing evidence‐based high‐intensity compound movements and high volume.^29^ However, our study included elderly individuals, many of which were undergoing radiation therapy, systemic treatment, and/or antiestrogen therapy. Additionally, all patients on study underwent at least a lumpectomy for their breast cancer, with the majority undergoing axillary lymph node resection, and many undergoing mastectomy, reconstruction, and axillary dissection. The improvements seen in strength and mobility in these individuals were without injury. Additionally, quality of life metrics were improved in these individuals, supporting the inclusion of oncologic patients with diverse treatment backgrounds in high‐intensity resistance training protocols.

Patient‐specific exercise oncology recommendations must be tailored to specific desired outcomes in the context of baseline functional status, comorbidities, cancer‐specific risk reduction, and prior and ongoing oncologic therapies. Among other exercise types, many BC survivors may uniquely benefit from resistance training due to [1] improved body composition and [2] improved functional mobility and balance. Regarding body composition, the present study showed significant increases in muscle mass with concurrent decreases in body fat, without significant changes in body weight. Within the BC population, the association of body mass index (BMI) to overall mortality, while significant (obese vs normal weight: RR 1.41, 95% CI 1.29–1.53),^30^ must be interpreted in the context of the prognostic association of sarcopenia in the same population (HR = 2.86, 95% CI, 1.67–4.89).^31^ Thus, an intervention focused on BMI alone, while accounting for the prognostic significance of excess body fat, may underestimate patient‐specific benefits associated with increased muscle mass. Changes in body composition parameters across ultrasound and InBody measurements appeared comparable. A recent meta‐analysis has revealed muscle mass increases of less than 1 lb, with fat loss of around 1 lb in breast cancer patients undergoing resistance training.^32^ We experienced larger reductions in adipose tissue and slightly larger increases in muscle mass gain in only 3 months. Additionally, the reduction in percent body fat of 2.9%–4.8% is substantial, particularly when compared to large dietary weight loss studies.^33^

As expected, bone mineral content (BMC) did not significantly differ after a 3‐month timeframe. While one might expect high intensity exercise to stimulate increased bone density, such effects within a population would likely require a longer timeframe and will be evaluated in subsequent longitudinal analyses. However, it remains notable that a decrease in median BMC was not observed despite an 80% prevalence of concurrent anti‐estrogen therapy.

Functional mobility and balance are of specific interest to BC survivors to mitigate the risk of falls and associated hip fracture in the context of anti‐estrogen therapy.^34^ The National Council on Aging estimates that 1 in 3 adults aged ≥65 years falls each year, and unintentional falls are the leading cause of nonfatal and fatal injuries in this population.^35^ The present data demonstrated concurrent increases in exercise performance and Y Balance test scores, as has been previously demonstrated among elderly and middle‐aged women.^36^ Functional movement scores, associated with decreased fear of falling and greater self‐perceived balance among older adults,^37^ similarly improved significantly after 3 months of resistance training. Concurrent increases in quality of life questionnaire scores may reflect similar changes in self‐perceived functional mobility. A recent randomized trial of BC survivors failed to demonstrate significant improvements in balance following a resistance training regimen^23^; however, resistance training on this trial involved machine‐based exercises performed at a volume and intensity which may be below the threshold necessary for improvements in strength and balance.^15^ Future studies employing linear progression of high‐intensity compound movements are needed to better define the correlations of Y‐balance and FMS testing to incidence of falls and associated trauma.

The present trial must be interpreted within the context of several limitations. First, it is difficult to ascertain the degree of selection bias that may result from the significant time and logistical requirements imposed by thrice‐weekly sessions up to 60 min in duration. Randomization to other activity‐based interventions might provide insight among what may be an especially motivated, high‐resource population. To this end, where resistance training methodologies remain heterogeneous across published reports, the authors recommend a minimum of 10 weekly sets of high‐intensity (≥80% 1RM) compound movements across major movement patterns (push, pull, hip hinge, squat, core activation) Second, possible contribution of nutrition, sleep, stress, and competing comorbidities among other parameters is beyond the scope of the present study. There is an inherent risk of type 1 error rate given the many different outcomes assessed via *t* tests. Additionally, attempting to increase intensity though assessment of number of repetitions “left in the tank” and RPE are inherently subjective, but this limitation further illustrates the importance of qualified personnel present to safely dose escalate the exercise regimen.

In summary, a 3‐month dose‐escalated resistance training regimen comprising high‐intensity compound movements dually supervised by oncologists and CSCS practitioners in accordance with NSCA guidelines appears safe with a high degree of adherence among breast cancer survivors, resulting in demonstrable improvements in body composition, metabolic parameters, strength increases, and patient‐reported quality of life. Future exercise oncology studies involving resistance training may similarly wish to employ similar methodologies, including at least 10 weekly sets at ≥80% 1RM intensity across compound movements.

## AUTHOR CONTRIBUTIONS

**David J. Carpenter:** Data curation (lead); formal analysis (lead); methodology (equal); validation (equal); writing – original draft (lead); writing – review and editing (equal). **Chris Peluso:** Data curation (equal); formal analysis (equal); investigation (equal); writing – review and editing (equal). **Christie Hilton:** Investigation (equal); project administration (equal); resources (equal); validation (equal); writing – review and editing (equal). **Frank Velasquez:** Conceptualization (equal); formal analysis (equal); writing – review and editing (equal). **Adam Annichine:** Conceptualization (equal); resources (equal); writing – review and editing (equal). **Krista Matsko:** Resources (equal); supervision (equal); writing – review and editing (equal). **Jared Rosenberg:** Conceptualization (equal); data curation (equal); formal analysis (equal); investigation (equal); methodology (equal); supervision (equal); writing – review and editing (equal). **Alexander K. Diaz:** Conceptualization (equal); data curation (equal); investigation (equal); methodology (equal); writing – review and editing (equal). **Parker Hyde:** Conceptualization (equal); methodology (equal); supervision (equal); validation (equal); writing – review and editing (equal). **Sushil Beriwal:** Data curation (equal); methodology (equal); writing – review and editing (equal). **Colin E. Champ:** Conceptualization (lead); data curation (equal); formal analysis (equal); funding acquisition (equal); investigation (equal); methodology (lead); project administration (lead); resources (equal); software (equal); supervision (equal); validation (equal); visualization (equal); writing – original draft (equal); writing – review and editing (equal).

## FUNDING INFORMATION

None.

## CONFLICT OF INTEREST STATEMENT

CEC receives income from books and lectures pertaining to nutrition and exercise.

## Supporting information


Data S1.

## Data Availability

The data that support the findings of this study are available from the corresponding author upon reasonable request.
