# Supplementary material for: EXERT‐BC: A pilot study of an exercise regimen designed to improve functional mobility, body composition, and strength after the treatment for breast cancer
Source: Cancer Med. 2024 Mar 16;13(5):e7001. doi: 10.1002/cam4.7001 (PMC10943368; doi:10.1002/cam4.7001)
Supplement: Supplementary file 1 — Data S1. [file CAM4-13-e7001-s001.pdf]

PROGRAM 1			
DATE			
LAZY BEAR			
DIAG. SIT KNEE			
BRETTZEL			
PULLOVER W/HAMSTRINGS			
WINDSHIELD			
BAND PNF			
SIDE ARM RAISE			
TWISTER			
ANKLES			
LAT STRETCH			
	EXERCISE	WEIGHT	REPS
A1	SPLIT SQUAT 3-4x8		
A2	SIDE PLANKS 20 s Each Side		
B1	BIRD DOG ROW 3x10		
B2	1-LEG GLUTE BRIDGE 3x10		
C1	1/2 KNEELING SHOULDER PRESS 3x10		
C2	BICEP DB CURLS 3x10		
HANGS OR BILATERAL PULLS			

PROGRAM 2			
DATE			
LAZY BEAR			
DIAG. SIT KNEE RAISE			
BRETTZEL			
PULLOVER W/HAMSTRINGS			
WINDSHIELD			
BAND PNF			
SIDE ARM RAISE			
TWISTER			
ANKLES			
LAT STRETCH			
	EXERCISE	WEIGHT	REPS
A1	GOBLET SQUAT 3-4x8		
A2	BAND PULL APARTS 3x10		
B1	HIP THRUST 12" plyo box 3x10		
B2	INCLINE DB PRESS 3x10		
C1	DB SKULL CRUSHERS 3x10		
C2	DB LAT RAISE 3x10		
HANGS OR BILATERAL PULLS			

PROGRAM 3			
DATE			
LAZY BEAR			
DIAG. SIT KNEE RAISE			
BRETTZEL			
PULLOVER W/HAMSTRINGS			
WINDSHIELD			
BAND PNF			
SIDE ARM RAISE			
TWISTER			
ANKLES			
LAT STRETCH			
	EXERCISE	WEIGHT	REPS
A1	HEX DL 3-4x8		
A2	TRX ROW 3x10		
B1	BOX STEP UP 3x10		
B2	PUSH UP 3X10 *W/KNEES/BOX *ASSIST W/BAR		
C1	SUIT CASE CARRY Turf distance x 3		
C2	DB HEEL TOES Turf distance x3		
HANGS OR BILATERAL PULLS			
